# The effectiveness of photobiomodulation therapy on inferior alveolar nerve injury: A systematic review and META-analysis

**DOI:** 10.1371/journal.pone.0287833

**Published:** 2023-08-10

**Authors:** Yongqing Ma, Miaomiao Yang, Xiaodong Chen, Weiguo Qu, Xiaofu Qu, Ping He

**Affiliations:** 1 Department of Oral and Maxillofacial Surgery, Dalian Stomatological Hosipital, Dalian City, Liaoning Province, China; 2 Department of Implantation, Dalian Stomatological Hosipital, Dalian City, Liaoning Province, China; 3 Department of Prosthodontics, Dalian Stomatological Hosipital, Dalian City, Liaoning Province, China; 4 VIP Clinic, Dalian Stomatological Hosipital, Dalian City, Liaoning Province, China; 5 Department of Oral Surgery, Dalian Stomatological Hosipital, Dalian City, Liaoning Province, China; Medical University of South Carolina, UNITED STATES

## Abstract

**Objective:**

The aim of this META-analysis was to evaluate the efficacy of photobiomodulation (PBM) therapy in the treatment of inferior alveolar nerve (IAN) injury due to orthognathic surgeries, extraction of impacted third molars and mandibular fractures.

**Methods and materials:**

A electric search was conducted by a combination of manual search and four electric databases including Pubmed, Embase, Cochrane library and Web of Science, with no limitation on language and publication date. Gray literature was searched in ClinicalTrials.gov and googlescholar. All retrieved articles were imported into ENDNOTE software (version X9) and screened by two independent reviewers. All analysis was performed using the REVMAN software (version 5.3)

**Results:**

Finally, 15 randomized controlled trials met the inclusion criteria for qualitative analysis and 14 for META-analysis from 219 articles. The results showed that PBM therapy had no effect on nerve injury in a short period of time (0-48h, 14 days), but had significant effect over 30 days. However, the effect of photobiomodulation therapy on thermal discrimination was still controversial, most authors supported no significant improvement. By calculating the effective rate of PBM, it was found that there was no significant difference in the onset time of treatment, whether within or over 6 months.

**Conclusions:**

The results of this META-analysis show that PBM therapy is effective in the treatment of IAN injures no matter it begins early or later. However, due to the limited number of well-designed RCTs and small number of patients in each study, it would be necessary to conduct randomized controlled trials with large sample size, long follow-up time and more standardized treatment and evaluation methods in the future to provide more accurate and clinically meaningful results.

## Introduction

Oral and maxillofacial surgery, such as local anesthesia, wisdom tooth extraction, implant implantation, orthognathic surgery, and rigid internal fixation of fractures, might cause trigeminal nerve injuries [1, 2]. Among them, the damage of IAN was the most common complication [3]. Secondary paresthesia may be temporary or permanent, but it could seriously affect the quality of life of the patient, such as drinking and eating, and might bite the lower lip due to sensory loss, some patients have neuropathic pain, unfortunately, only part of these patients could be self-remission [4, 5]. Therefore, appropriate treatment measures should be taken to treat and alleviate the abnormal and missing nerve sensation. As the classifications for the damage of nerves, there are many methods to treat nerve injuries, including medications, electrical stimulation, acupuncture, physical therapy, microneurosurgey, etc [6–8]. The degree of nerve injuries was classified into three types by Seddon: neurapraxia, axonotmesis and neurotmesis [9]. If neurotmesis occurs, a microsurgical anastomosis or nerve transplantation is required. Most of the nerve injuries we encountered are mainly combinations of neurapraxia and axonotmesis, the inclined treatments are conservative and non-invasive ones, which are more acceptable to patients [10].

In the past decades, some scholars have reported that low-intensity laser can stimulate and treat nerve injury without thermal effects in the purpose of relieve pain, promote tissue repair and anti-inflammation [11–17]. Although the mechanism has not reached an agreement yet, it has been proved that low-intensity laser irradiation would make an increase in beta-endorphin production, neurotransmission and flow, thus allowing for the drainage of substances from inflammation and its consequent regulation, in addition accelerated tissue repair, bone regeneration, and the re-establishment of neural function [18, 19]. Furthermore, it can repair damaged DNA and promote enzyme activity, induce therapeutic cascade and photochemical regulation, and activate the body’s immune system [20]. There is also hypothesis that laser radiation stimulates tissue reinnervation by penetrating axons or in adjacent Schwann cells to stimulate the metabolism of damaged neurosensory tissue and the production of proteins associated with the growth of uninjured adjacent nerves [28].

However, there are few reports about low-intensity laser treatment of IAN injury, and the high-quality RCT is even less, and the evaluation methods are different and the sample size is generally small. In addition, previous systematic reviews and META-analyses have either outdated or focused on a specific cause of injury. To our knowledge, the main factor affecting the repair outcome is the extent of the injury [21–23]. Therefore, a more comprehensive and updated meta-analysis is needed to explore the effectiveness of PBM therapy on IAN injury.

## Materials and methods

### Protocol and registration

The review protocol has been registered on PROSPERO (CRD42023397943).

This review was performed in accordance with both the recommendations from the Cochrane’s Handbook for Systematic Reviews and the Preferred Reporting Items for Systematic Reviews and Meta-Analysis for Protocols (PRISMA guidelines), aim to evaluate whether the low intensive laser or LED therapies could be an available treatment modality for the sensory recovery after lower alveolar injures [24, 25].

### Eligibility criteria

Original articles were selected based on the PICOS (Population Interventions Comparison Outcomes Study design) models, regardless of language and publication date.

The PICOS model for this systematic review was as follows: population (patients who underwent IAN injuries due to wisdom teeth extraction, orthognathic surgery, implantation and rigid internal fixation of fractures ), intervention (lower intensity laser therapy ), comparison (other side of lower jaw as placebo, control group of participants who received no treatments ), outcome (laser therapy result in recovery, alleviation or no improvement of neurosensory disorders ), study design (randomized controlled trials).

#### Search strategy

A systematic search was performed in the following electronic databases: PubMed, Cochrane Central Register of Controlled Trials (CENTRAL), Cochrane library, Embase, and Web of Science, with no limitation on language, from the start of the database to February 2023 by limited in title, key words and abstracts. The subject words are retrieved from MESH subject list query and combined with free words query, so as to search completely. Manual screening was conducted through reading the references of included articles and related reviews for additional relevant publications. Gray literature was searched in ClinicalTrials.gov and googlescholar. Additionally, the reference list of published systematic reviews was cross-checked for additional eligible studies.

The search strategy in databases is presented in Table 1.

**Table 1:** Complete search strategy.

Searching phrase	(Low-Level Light Therapy OR Light Therapies, Low-Level OR Light Therapy, Low-Level OR Low Level Light Therapy OR Low-Level Light Therapies OR Therapies, Low-Level Light OR Therapy, Low-Level Light OR Photobiomodulation Therapy OR Photobiomodulation Therapies OR Therapies, Photobiomodulation OR Therapy, Photobiomodulation OR LLLT OR Laser Therapy, Low-Level OR Laser Therapies, Low-Level OR Laser Therapy, Low Level OR Low-Level Laser Therapies OR Laser Irradiation, Low-Power OR Irradiation, Low-Power Laser OR Laser Irradiation, Low Power OR Low-Power Laser Therapy OR Low Power Laser Therapy OR Laser Therapy, Low-Power OR Laser Therapies, Low-Power OR Laser Therapy, Low Power OR Low-Power Laser Therapies OR Low-Level Laser Therapy OR Low Level Laser Therapy OR Low-Power Laser Irradiation OR Low Power Laser Irradiation OR Laser Biostimulation OR Biostimulation, Laser OR Laser Phototherapy OR Phototherapy, Laser) AND (injur* OR damage OR sensory change OR neurosensory disturbance OR neurosensory deficits OR neurosensory disorder) AND (Mandibular Nerve OR Alveolar Nerve, Inferior OR Inferior Alveolar Nerve OR Inferior Alveolar Nerves OR Nerve, Inferior Alveolar)

### Study selection

All retrieved articles were imported into ENDNOTE software (version X9). After removing duplicate articles, the titles and abstracts were pre-screened by two reviewers independently, and eligible articles were selected for further screening by full-text reading. Any disagreement between the two readers was resolved through discussion, consensus, or consultation by a third author. After that, we excluded the irrelevant studies based on the inclusion and exclusion criteria and the reason for exclusion was listed for further reporting.

### Data extraction

From the included studies, the following data was extracted using a predetermined information extraction table: the publication date, design of the study, sample size, sex and age of the participants, comparison(s), the etiology of the injury, time between injury and treatment, follow-up period, laser wavelength, energy density, radiation time for each point, total number of therapeutic sessions, time of laser application and types of neurosensory tests.

Meanwhile, if data were insufficient or only expressed graphically, numerical values were acquired by contacting the authors, and if no response was received, a digital ruler software was used to measure graphical data (Available from https://automeris.io/WebPlotDigitizer/). When studies reported outcomes at multiple time points, data from similar time-points of different studies were pooled for meta-analysis. The data extraction was performed independently by two investigators and any disagreements were resolved by discussion.

### Quality assessment

Two reviewers independently evaluated the risk of bias for RCTs in this research using Cochrane Collaboration’s assessment tool [26]. Seven domains of bias were evaluated: (1) random sequence generation (selection bias), (2) allocation concealment (selection bias ), (3) blinding of participants and personnel (performance bias), (4) blinding of outcome assessment (detection bias), (5) incomplete outcome data (attrition bias), (6) selective reporting (reporting bias), and (7) others (follow-up period, baseline).

### Statistical analysis

Because only tests such as two-point discrimination, general VAS scores, and contact tests (Semmes-Weinstein monofilaments) have sufficient data, these continuous outcome variables and dichotomous variables (post-treatment improvement rate) were extracted for META-analysis. All data were analyzed using REVMAN (version 5.3) software to determine pooled effects. Mean difference with 95% confidence interval was used and P < 0.05 was considered for a significant difference. Heterogeneity was tested using I^2^ statistics. I^2^ < 50% at the level of α > 0.10 indicated a lack of heterogeneity across the studies.

For continuous variables, the standard mean difference (SMD) was considered due to the numerically different data. The fixed-effect model was applied if the P value related to the heterogeneity was more than 0.10. Otherwise, the random-effects model was used.

## Results

### Eligibility of the studies

**Fig 1:**
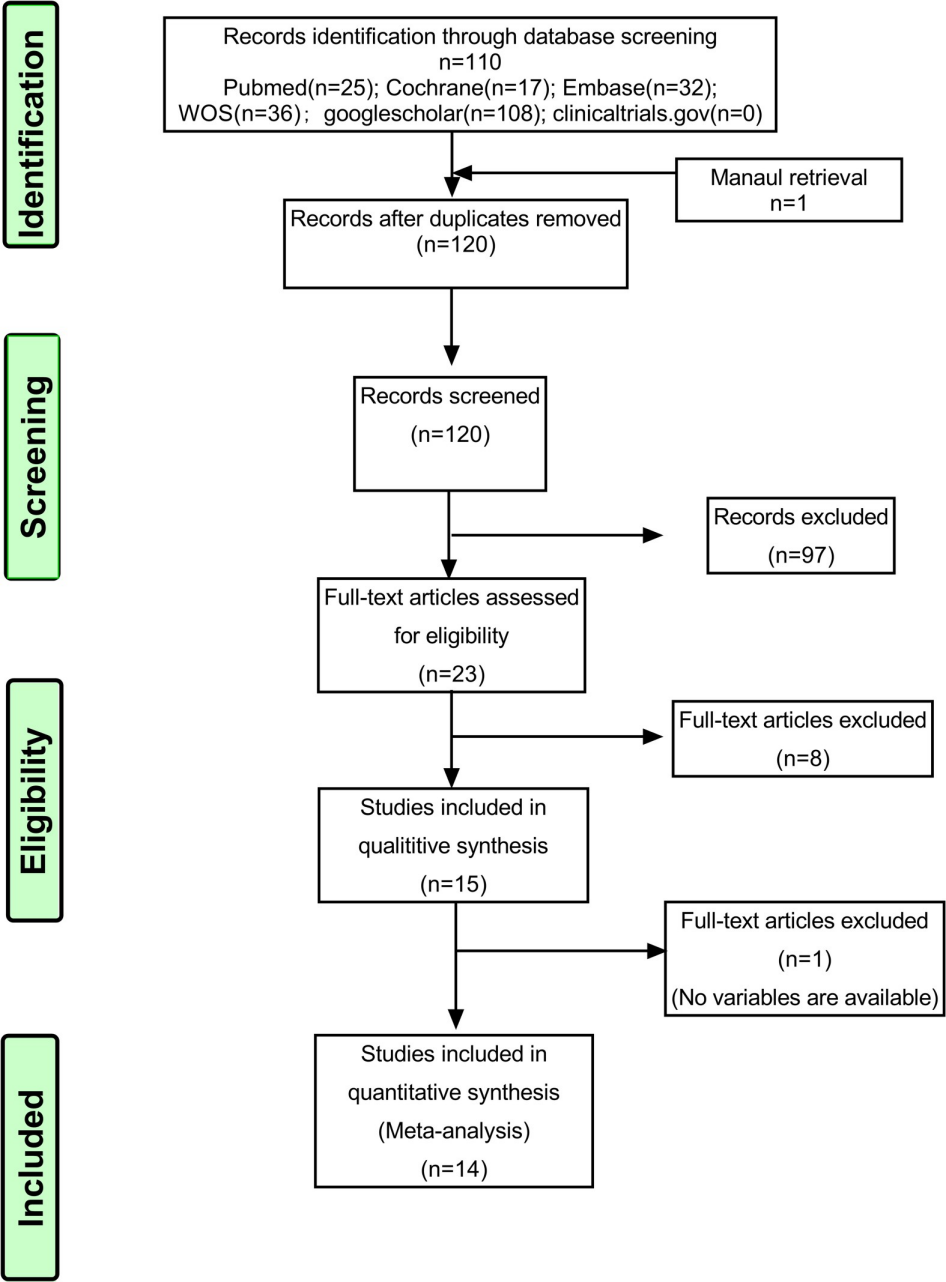
Flow chart of the literature search and selection.

After a thorough search, a total of 219 articles were imported into ENDNOTE software. After removing 99 duplicates articles, the titles and abstracts of the remaining 120 articles were assessed and the articles of animal experiment, review, case report, irrelevant contents or those full-text and abstract couldn’t be obtained were excluded. A total of 23 articles were retrieved. Through reading the full text comprehensively, 15 articles were selected for systematic review after excluding 8 articles which were not the randomized controlled trials or the data of tongue nerve injuries was mixed in the results. Due to the lack of numerical data in one of the papers [27], finally, the data from 14 articles were assessed by META-analysis [11–15, 28–36] (Fig 1).

### Study characteristics

This review included articles from 1996 to 2022. The number of patients involved ranged from 10 to 52, and 6 studies had the split-mouth design [11, 15, 28, 34, 35, 36]. The etiologies of IAN injuries included extraction of impacted third molars, orthognathic surgery and mandibular fractures. In most studies, either the control groups or the control sides used off-switch or no-treatment as a placebo, only one study used Mecobalamin as the control group.

The shortest time to start treatment was immediately after operation [11, 27, 34], and the longest was 177 months after anesthesia [13]. The frequency of laser treatment ranged from 5 to 12 times, with the shortest interval of 1 day and the longest period of 3 to 4 weeks [12, 28]. Laser wavelengths were between 780nm and 980nm, and some studies had used LED in combination or alone between 632nm and 850nm [12, 31, 34]; the exposure time at each point or site was between 28 seconds and 20 minutes, with the energy density range from 1.5J/cm^2^ to 157.5J/cm^2^. Also, combined use of intraoral and extraoral radiation was conducted in some studies, with different wavelengths, radiation densities and energy sources.

The assessments of neurosensory recovery included visual analog scale for general sensitivity (VAS), two-point discrimination (TPD), pinprick, thermal discrimination, pain discrimination, directional discrimination (brush stroke) and contact detection (Semmes-Weinstein monofilament test).

Characteristics of the included articles are presented in Table 2.

**Table 2:** Summary of included studies.

**(Part 1)**								
Study	Sample size (T/C)	Gender(T/C)	Mean age(T/C)	Etiology of injury	Timing of repair	Follow-up period	Wavelength(nm)	Energy density
2020 Sharifi, R.	18/18(split)	4/14	23±5	SSRO	1 day ahead	1 months	980	12 J/cm^2^
2022 Santos, V. P. D.	22/20	No difference	29.4	mandibular fractures	1 day later	6 months	660∼850	7.64 J
2019 Santos, F. T.	20/20(split)	7/13	35.6 ± 11.6	SSRO	within 30 days or 6 months later	1 year	780	157.5 J/cm^2^
2020 Qi, W.	10/10	Not mentioned	No difference	Tooth extraction	24 h after extraction	1 month	808	3 J/cm^2^
2017 Mohajerani, S. H.	10/10	5/5 3/7	24.1± 4.6/22.8 ± 3.6	SSRO	1 day after	6 months	632–810	Laser: 5J/cm^2^ LED: 2J/cm^2^
2018 Guarini, D.	33/9	13/20 2/7	25.8/29.8	SSRO	1 day after	2 years	810–820	27J
2014 Gasperini, G.	10/10(split)	0/10	30	SSRO	immediately after surgery	2 months	660/ 789/780	5/30/70J/cm^2^
2014 Fuhrer-Valdivia, A.	16/14	5/11 4/10	21.5±8 /23± 5	SSRO	immediately after surgery	6 months	810±20	32J/cm^2^
2018 Esmaeelinejad, M.	20/20	18/22	26.52 ± 3.78	SSRO	immediately after surgery	12 months	810	8.4 J/cm^2^
2017 Eshghpour, M.	16/16(split)	5/11	23.1±4.4	SSRO	1 day after	2 months	660/810	1.5/7 J/cm^2^
2017 Astrid Virginia Buysse	12/12(split)	6/6	30	orthognathic surgery	48h after surgery	6 weeks	808	100 J/cm^2^
2022 Salari, B.	26/26	6/46	35.69±13.44/36.23 ± 11.51	mandibular fractures	6 months later	6 months	810	12–14 J/cm^2^
1996 Khullar, S. M. B. P.	6/7	Not mentioned	Not mentioned	SSRO; Tooth extraction	6 months later	36∼69	820	550mW/cm^2^
1996 Khullar, S. M. E. B.	8/5	4/9	35.7	SSRO	2 years later	63 days	820	550mW/cm^2^
2020 Esteves, P. F. P	12/12(split)	6/6	30	SSRO	48h after surgery	6 weeks	808	157.5 J/cm^2^
**(Part 2)**								
Study	Radiation time	Days of laser therapy	Types of neurosensory tests					
2020 Sharifi, R.	60s	0, 1, 3, 7, 14, 21, 28	VAS, BS, TPD, PD, TD					
2022 Santos, V. P. D.	20 min	every day for 6 months	PP, TD					
2019 Santos, F. T.	90s	3-to-4 weeks intervals	SWMF					
2020 Qi, W.	188s	once every two days between 2 to 14 days	VAS, TPD					
2017 Mohajerani, S. H.	90s	1 , 2, 3, 7, 14, 28	VAS, BS, TPD, PP, TD, SWMF					
2018 Guarini, D.	90S	1, 2, 3, 5, 10, 14, 21, 28	VAS, TPD, PD, TD					
2014 Gasperini, G.	10/20/ 40s	0, 1, 2, 3, 5, 7, 9, 11, 13, 15, 17, 19, 21, 23	TPD, PP					
2014 Fuhrer-Valdivia, A.	90s	1,2,3,5,10,14,21,28	VAS, BS, TPD, TD					
2018 Esmaeelinejad, M.	28s	0, 1, 2, 3 then every other day for two weeks	TPD, TD, PP, SWMF					
2017 Eshghpour, M.	10s	1, 2, 3 and then twice a week for 3 weeks	TPD					
2017 Astrid Virginia Buysse	28s	2 and two sessions per week	VAS					
2022 Salari, B.	30s	twice a week for 6 weeks	light touch, PP, TPD, TD, EPT					
1996 Khullar, S. M. B. P.	85s	20 sessions between 36 and 69 days	TD, SWMF					
1996 Khullar, S. M. E. B.	85s	20 sessions between 20 and 63 days	VAS, SWMF					
2020 Esteves, P. F. P	28s	2rd day and then two sessions per week	VAS					

T = treatment group; C = control group; TD = thermal discrimination; VAS = visual analog scale test; SWMF = Semmes-Weinstein monofilament test; TPD = two-point discrimination; PP = pinprick; PD = pain discrimination; EPT = electric pulp test; BS = brush stroke

SSRO = sagittal split ramus osteotomy

### Risk of bias within studies

**Fig 2:**
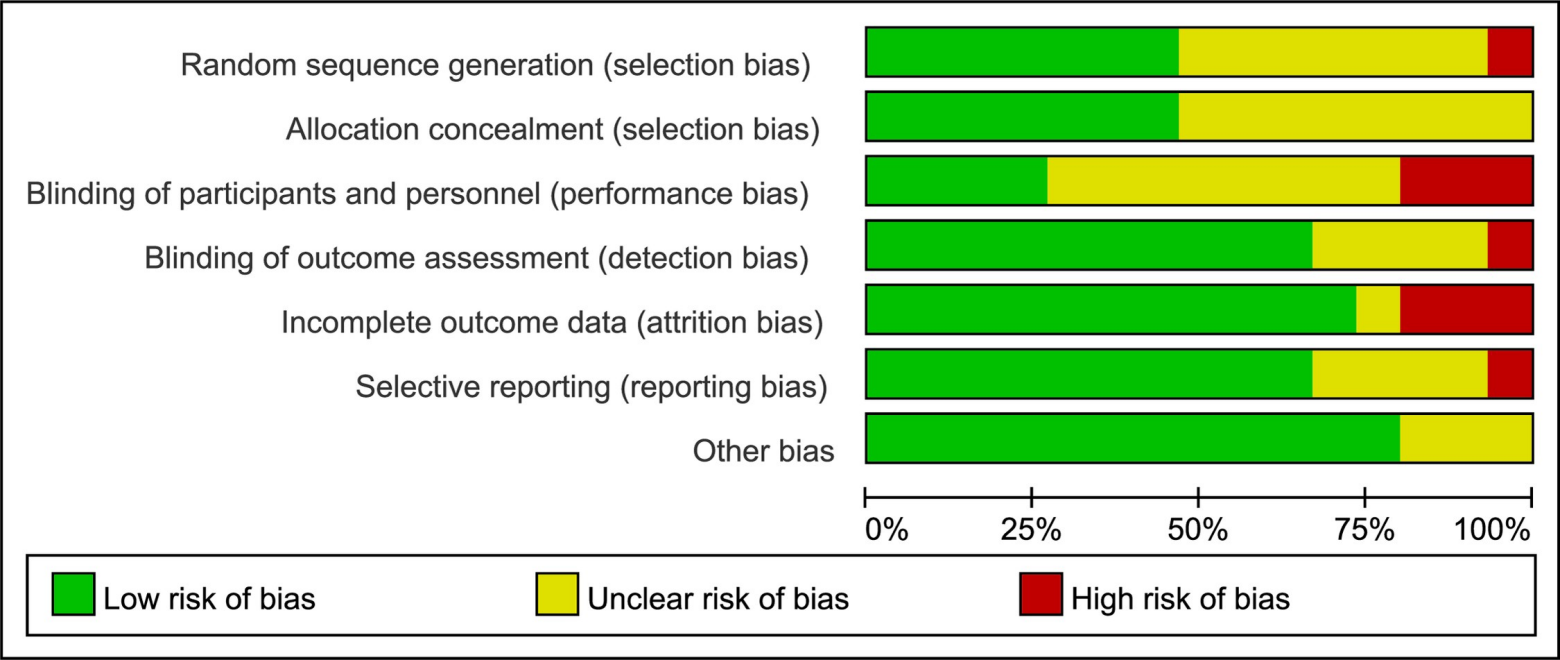
Risk of bias graph.

**Fig 3:**
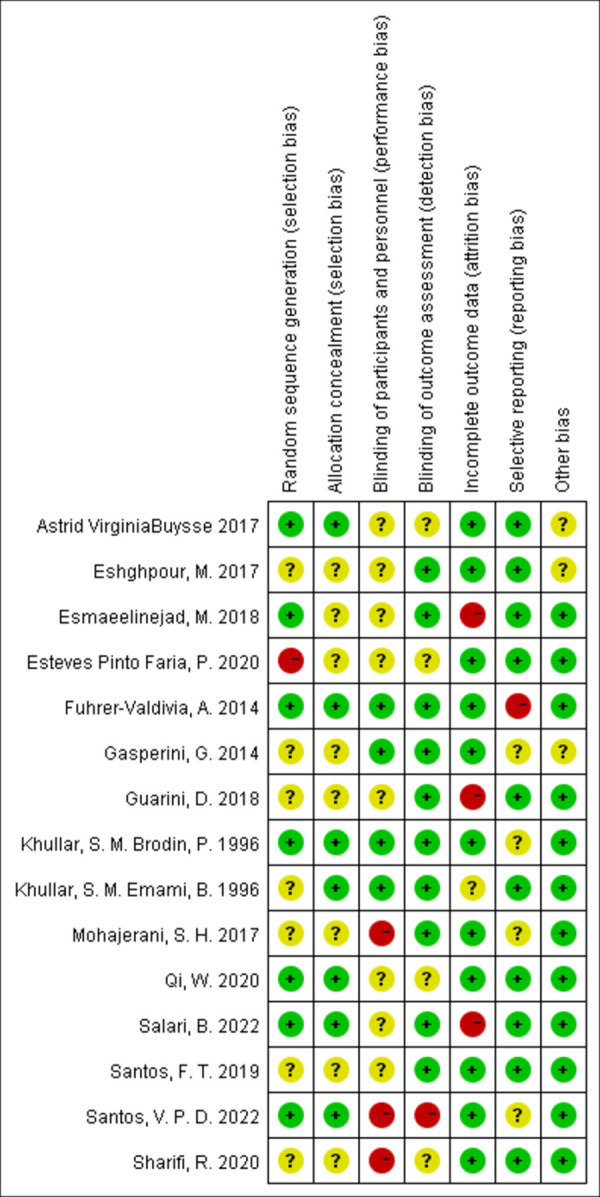
Risk of bias summary.

The summary and graph of risk of bias assessment for included RCTs are illustrated in Figs 2 and 3. The risk of bias for most studies was high, which was mainly reflected in randomization, allocation concealment and blinding of participants and practitioners. Some literature mentions randomization, but does not address specific methods of randomization and allocation concealment, such as random number tables, envelopes, and so on [11, 15, 27, 28, 31, 33, 34]. Although some studies could blind the practitioner, only four experiments could blind the participants at the same time because of the visibility and noisiness of the laser radiation [13, 14, 32, 34]. The specific methods included turning off the switch, or confusing the participants’ perception with eye masks or earplugs.

### Data synthesis

#### 1. Visual anglog scale for general sensibility (VAS)

**Fig 4:**
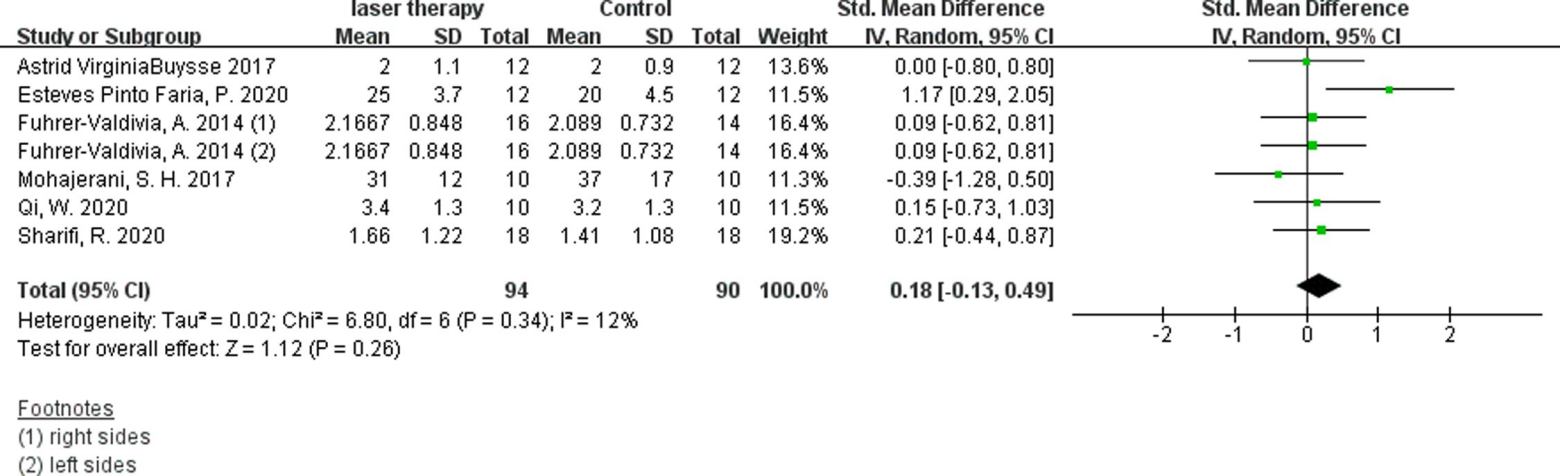
Forest plots of visual anglog scale for general sensibility showing the effectiveness of PBM therapy within 48 hours.

**Fig 5:**
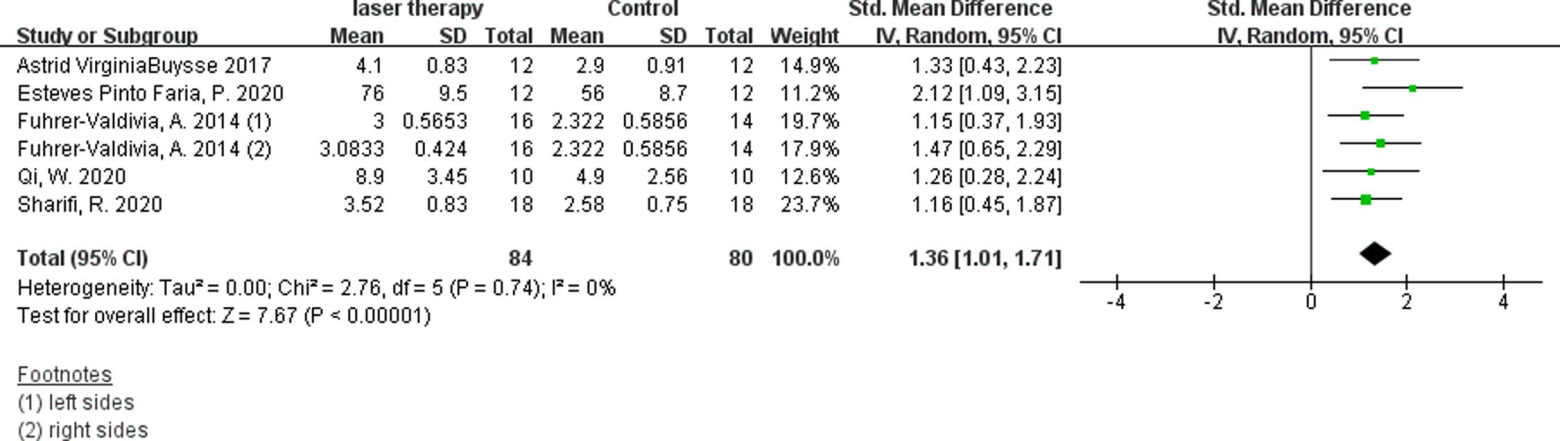
Forest plots of visual anglog scale for general sensibility showing the effectiveness of PBM therapy in 30 days.

**Fig 6:**
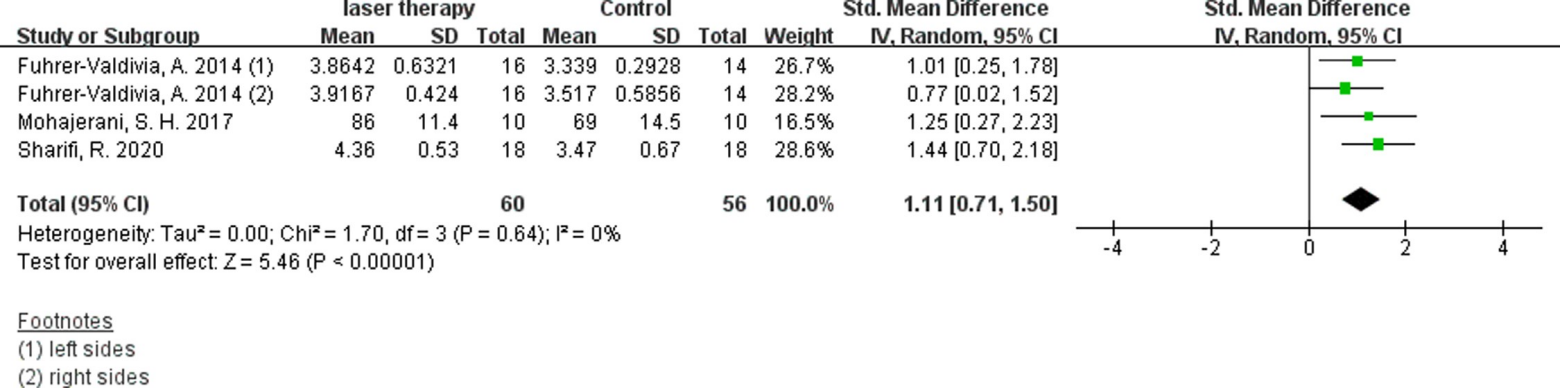
Forest plots of visual anglog scale for general sensibility showing the effectiveness of PBM therapy in 60 days.

Six studies evaluated the efficacy of low-intensity laser therapy using VAS and available digital data were obtained [11, 14, 30, 31, 35, 36]. According to the synthesis data, the short-term postoperative laser treatment within 48 hours had no significant effect (Fig 4, SMD 0.18, 95% CI: −0.13 ∼ 0.49, I ^2^ = 12%, no obvious heterogeneity), but it was significantly effective in 30 days(Fig 5, SMD 1.36, 95% CI: 1.01 ∼ 1.71, I ^2^ = 0%, no heterogeneity)and 60 days(Fig 6, SMD 1.11, 95% CI: 0.71 ∼ 1.50, I ^2^ = 0%, no heterogeneity) after the treatment. The results of Fuhrer-Valdivia A et al. are reported randomly as the left and right sides respectively, so the data are inputed separately. Moreover, this study only provides the median and interquartile range, and the mean and standard deviation are calculated by means of Luo et al. [37] and Wan et al. [38].

#### 2. Two-point discrimination test (TPD)

**Fig 7:**
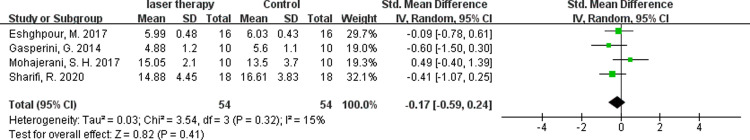
Forest plots of two-point discrimination test showing the effectiveness of PBM therapy within 48 hours.

**Fig 8:**
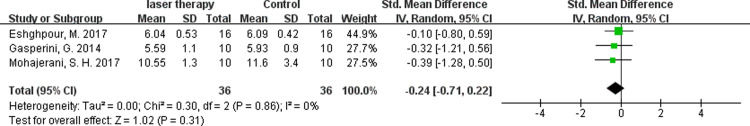
Forest plots of two-point discrimination test showing the effectiveness of PBM therapy in 14 days.

**Fig 9:**
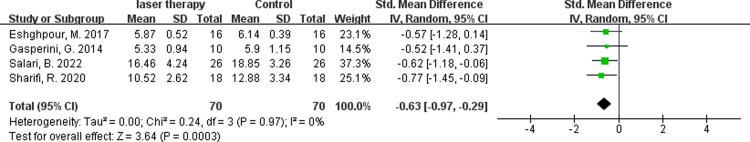
Forest plots of two-point discrimination test showing the effectiveness of PBM therapy in 30 days.

**Fig 10:**
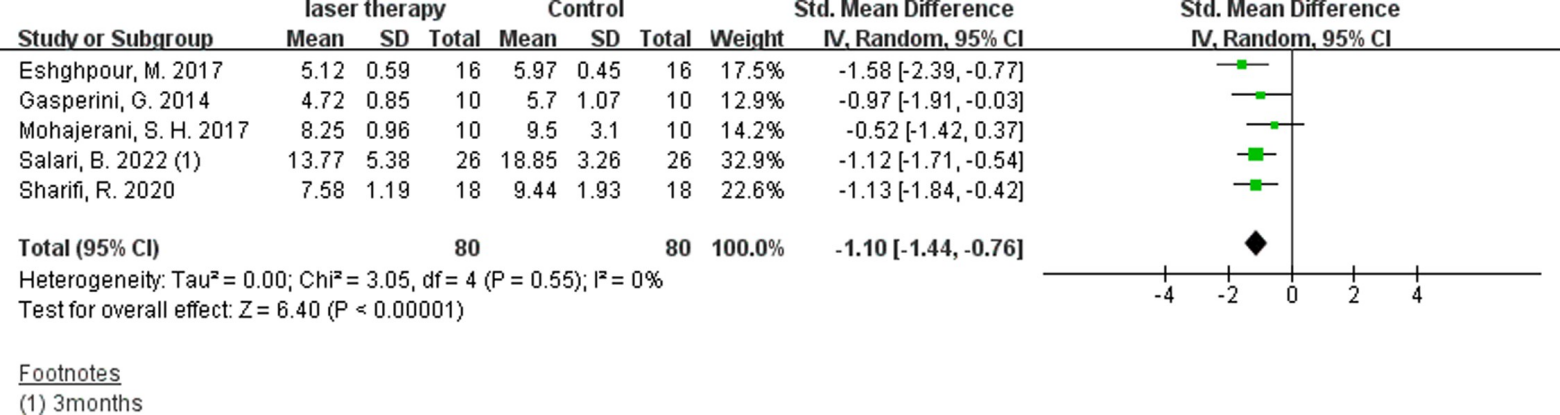
Forest plots of two-point discrimination test showing the effectiveness of PBM therapy in 60 days.

5 studies with two-point discrimination evaluations could extract available data[11, 15, 29, 31, 34]. In the test, the minimum distance the patient could distinguish between two separate stimuli points with a relatively sharp tip was recorded. There was no significant effect within 48 hours(Fig 7, SMD −0.17, 95% CI: −0.59 ∼ 0.24, I ^2^ = 15%, no obvious heterogeneity) and 14 days after treatment(Fig 8, SMD −0.24, 95% CI: −0.71 ∼ 0.22, I ^2^ = 0%, no heterogeneity). But the data of 30 days(Fig 9, SMD −0.63, 95% CI: −0.97∼ −0.29, I ^2^ = 0%, no heterogeneity) or 60 days(Fig 10, SMD −1.1, 95% CI: −1.44 ∼ −0.76, I ^2^ = 0%, no heterogeneity) after operation showed that the nerve recovery of the experimental group was obviously better than that of the control group after the radiation of low intensity laser.

#### 3. Contact detection (Semmes-Weinstein monofilaments test)

**Fig 11:**
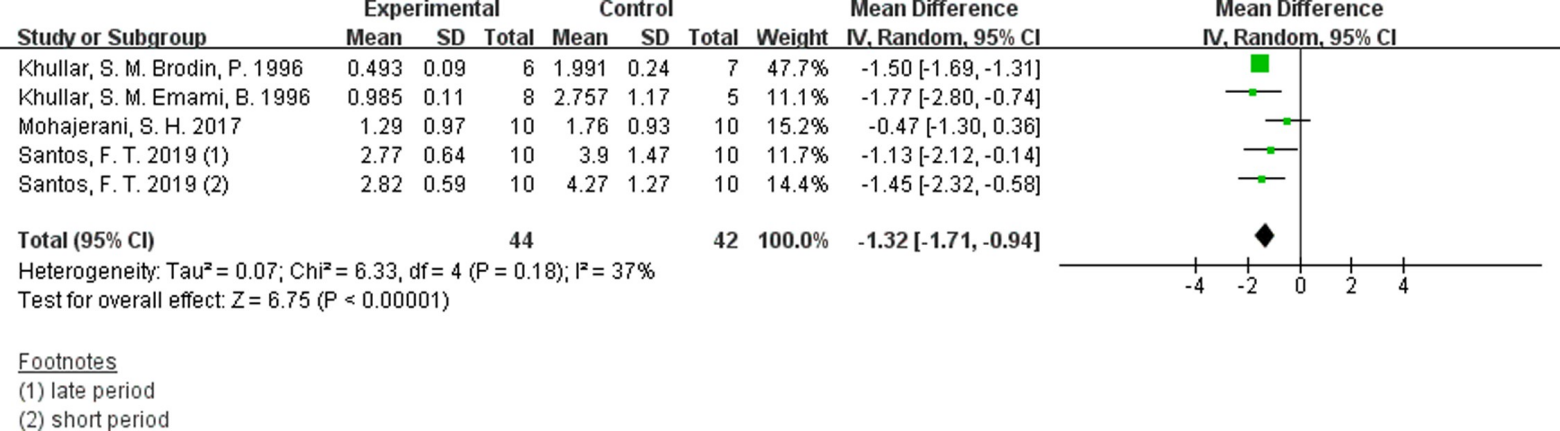
Forest plots of Semmes-Weinstein monofilaments test showing the effectiveness of PBM therapy.

In this test, the monofilaments were of constant length but different diameter, which were placed on the skin in each point until they were bend when specific load value was reached. The recognized size of the filament necessary to elicit a sensory response was recorded in grams. Santos et al’s study was separated into treatment groups within 30 days and treatment groups over 6 months according to the timing of repair, so it was divided into two separate researches when data was imported [28]. From the results from 5 studies, we can see that the skin or mucosa sensory recovery of experimental group is significantly better than the control group (Fig 11, SMD −1.32, 95% CI: −2.32 ∼ −0.58, I ^2^ = 37%, no obvious heterogeneity) [13, 28, 31, 32].

#### 4 Recovery percentage

**Fig 12:**
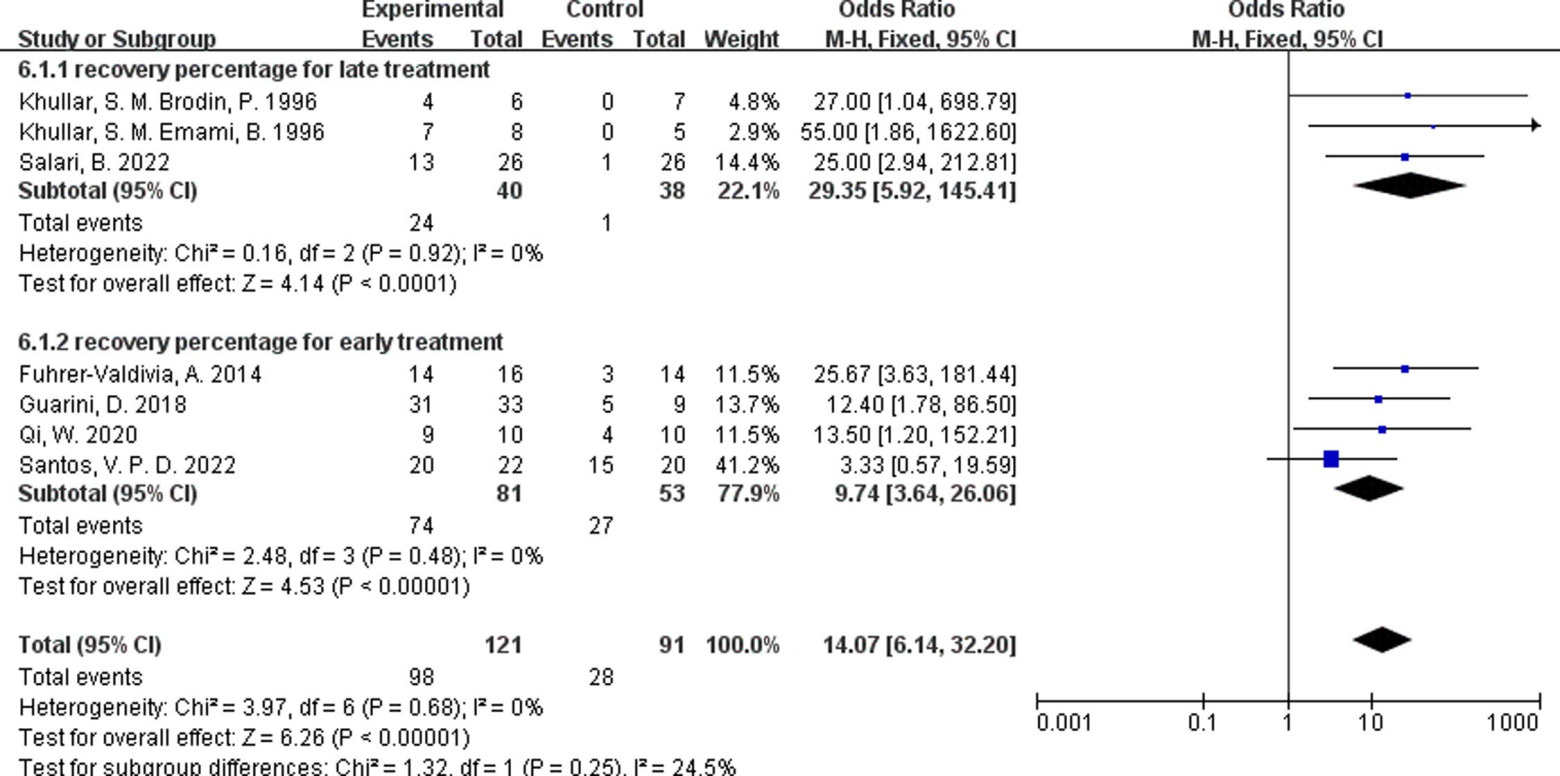
Forest plots of recovery rate for PBM therapy.

In some literature, the recovery rate is given directly [30, 31], and in others, the recovery rate of low intensity laser therapy can be calculated by the number of patients who had significant recovery evaluated by neurosensory evaluation tests such as TPD, VAS or contact detection, and so on [12–14, 32, 33]. By comparing the results of three studies with the onset of repair after six months and four studies treated within six months, there was no heterogeneity in data, but no significant difference in recovery rates between early and late repair(Fig 12, I ^2^ = 0, P = 0.25 ).

#### 5. Other neurosensory evaluations

Other sensory tests, such as temperature, pinprick, and direction discrimination, were not available or could not extract effective data for META-analysis. As reported by Sharifi, R et al. and Santos, V.P.D et al., the treatment group was significantly superior to the control group at 30 days for thermal discrimination, but the pooled data were not adopted because of high heterogeneity.

**Fig 13:**
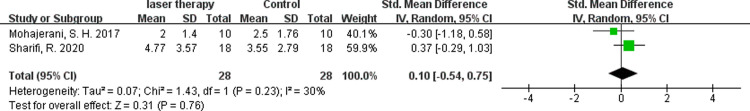
Forest plots of brush stroke test showing the effectiveness of PBM therapy within 48 hours.

**Fig 14:**
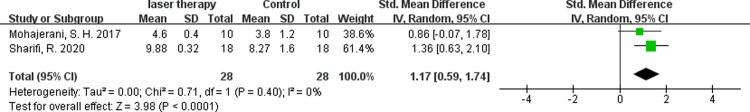
Forest plots of brush stroke test showing the effectiveness of PBM therapy in 60 days.

According to Sharifi R and Mohajerani SH [11, 31], there was no difference in the recovery of direction discrimination between the experimental and control groups one day after therapy (Fig 13, SMD 0.1, 95% CI: −0.54 ∼ 0.75, I ^2^ = 30%, no obvious heterogeneity), but after 60 days, the experimental group was significantly better than the control group (Fig 14, SMD 1.17, 95% CI: 0.59 ∼ 1.74, I ^2^ = 0, no heterogeneity). As for pain discrimination, although this test was mentioned in many articles, but only two of them could extract valid data, which proved that there was significant relief of pain after 30 days post-treatment [11, 12]. Anyway, the results were not adopted because of high heterogeneity (I ^2^ = 92%).

## Discussion

### Summary of evidence

The aim of this META-analysis was to evaluate the efficacy of PBM in the treatment of IAN injury. In accordance to previous literature, the sort of real objective evaluation methods should refer to tests such as trigeminal evoked potential, electrical thermography, electromyography, so most of the studies included in our review have used subjective tests [39]. Because of the differences in the methods of neurosensory evaluation, only the more widely used detection methods that can extract continuous variables are applied for quantitative analysis, such as VAS, two-point discrimination, contact experiment and direction discrimination, from which continuous variables were extracted for META-analysis. Through systematic review of 15 studies and META-analysis of 14 studies, it showed that low-intensity laser or LED had a significant therapeutic effect on IAN injuries caused by various reasons, capable of accelerating the rate or extent of recovery.

In the included studies, the etiologies of IAN damage were mostly sagittal split ramus osteotomy, only two studies for wisdom tooth extraction and two for mandibular fractures. Lesions due to impacted third molars extraction were mostly excluded because they were not RCTs or could not isolated from data mixed with lingual injuries [16, 17, 40–43]. In addition, Qi W et al’s article was the only one that used medicine therapy as a control (mecobalamin) instead of placebo. It is worth mentioning that the laser treatment method of this study is also different from the others, which put the probe into the post-extraction socket [30].

There are at least three primary modes of sensation recognized by peripheral terminal nerve endings of sensory axons. These are mechanoception, nociception, and thermoception, in which Aα and Aβ fibers responsible for mechanoception (touch) and Aδ and C fibers, responsible for pain and temperature [32, 43, 44]. The damage and recovery of IAN were evaluated mainly by VAS, TPD, pinprick, cold or warm discrimination, brush stroke and Semmes-Weinstein monofilament test.

As assessed by VAS and two-point discrimination tests, the initial use of PBM therapy did not benefit in the short period (within 48 hours and 14 days), but promising results could be observed more than 30 days later, which performed an beneficial effects on the regeneration of mechanoreceptors.

Detailed touch sensation, which is mediated by Aα and Aβ fibers, was evaluated by Semmes-Weinstein monofilament test. The results showed that PBM therapy could significantly accelerate and improve the recovery of superficial tactile sensation.

As for the improvement of temperature discrimination, there are controversies among different studies. Some literature supports that the recovery of thermal discrimination was statistically significant [12, 30]. However, Sharifi, R et al. reported the experimental group is obviously better than the control group at 30 days, but at 60 days, there is no difference between the two groups[11]. Mohajerani, S. H. et al. found that temperature perception returned to normal 7 days after surgery in both groups [31]. In other five studies, there was no significant difference between the two groups in temperature discrimination after treatment [13, 14, 29, 32, 33]. According to the authors, the thermoreceptor might returned to their previous state faster than other receptors [11]. On the other hand, warm sensation is thought to be mediated by specific afferent C-fibers and cold sensation by A-δ fibers. This suggests that laser therapy did not affect A-δ or C-fibers, which regulate the recovery of peripheral thermoreceptors [13, 45].

Six studies were assigned with a split-mouth design, all of which were performed with SSRO, with one side treated with laser and the other as a placebo group. Through this design, the anatomic differences of patients and the differences of operators’ operation can be maximally avoided.

The energy source mentioned in this review were mostly GaAlAs diode laser, but some studies have used or combined light-emitting diode (LED) as a source of energy, which have the advantages of producing little heat, more comfortable, less expansive and lightweight equipment, so patients can take home their for self-use conveniently and safely [12, 31, 34].

There are two possible ways for IANs to recover from injury, one is through the regeneration of nerve fibers after Wallerian degeneration, and the other is the collateral reinnervation by means of ingrowth from adjacent intact nerves [32]. Some scholars believed that laser therapy exerts its effect by increasing the release of a trophic factor within the area being treated and then stimulating colateral reinnervation from the other nonreactive adjacent tissues [46–48]. Others proposed the possible mechanisms of laser radiation on neurosensory regeneration related with the reduction of prostaglandins, acute phase inflammatory factors like TNF-α, IL-1 and interstitial metalloproteinase [49] and the synthesis of growth factors [50].

In this review, a subgroup analysis was performed to compare the differences in efficiency between patients treated within six months and those treated after six months, by extracting and calculating the number of patients who were obviously improved after laser treatment. To our surprise, there was no significant difference in the recovery rate. This is contrary to the view of some studies [23, 28], which means that it would never too late to treat the peripheral nerve injuries.

### Limitations and future directions

Of all the biases, publication bias is the most difficult to avoid. Because our analysis can only include published data and statistically significant or positive results are more likely to be submitted and published, it is conceivable that our estimates of effect size reported here might be overstated. In the meanwhile, there were 3 articles in the gray literature retrieved through googlescholar, whose titles seemed to be relevant, but the abstract and full text were not available, which may also increase the bias. Unfortunately, the amount of literature included for each outcome variable was insufficient, ranging from 2 to 7 respectively. If the included studies was greater than 10, sensitivity analysis could be conducted through funnel plot, which was qualitative analysis of publication bias, or quantitative analysis was done through trim and filling method, and so on.

Among the limitations of the reviewed papers, we can refer to the small sample size of the studies, shortness of follow up period and the large amount of heterogeneity in laser settings, such as the dissimilarities in the wavelength, energy density, irradiation time and treatment frequency, and the timing of repair, etc. On the other hand, there is no uniform standard for evaluation measurement tools for sensory impairment which can influence the comparability of the results from studies, even if the same evaluation method, the outcome variables are given in various forms, either the recovery rate or the numerical value.

If these problems cannot be solved, it will be difficult to obtain accurate and meaningful results. Therefore, it would be necessary to develop a standardized protocol to produce more meaningful experimental results that better serve the clinical treatment.

First of all, with the implementation of RCT experiment, it is better to use specific methods such as random number table to ensure random allocation. At the same time, in the process of laser treatment, we need blind not only the participants but also the examiner. For example, the participants were blinded by means of eye mask, ear plug and laser which noises without energy and the practitioner did not know the group and whether the laser was effective. Then the therapeutic effect was evaluated by another trained person who also did not know the allocation. In addition, for the establishment of control group, it is better to act with a split-mouth design for orthognathic surgery. However, more RCT studies should be carried out on nerve lesions via impacted wisdom teeth, implantation, and mandibular fractures to enrich the available literature and provide theoretical basis for higher quality research in future.

Secondly, with the parameters of laser/LED irradiation, most research has used 800-900nm wavelengths for light-emitting diode laser and 660nm for LED. The optimal time of laser irradiation was 90 seconds for each point. As for energy density, there are currently two general approaches, long intervals for large dose or short intervals for small dose. For example, treatment interval should be slightly longer if energy density is greater than 100 J/cm^2^ each time, but if the density is around 10J/cm^2^, it can be more frequent for once a day or every other day. Further research is needed to determine which approach is better [51]. Concerning the timing of treatment, it was hard to exactly evaluate the extent of nerve damage and the transient neurapraxia of the nerve might recover on its own between 1 and 6 months after impairment. Therefore, the best time for treatment should be at least 30 days after injury. As for the frequency of treatment, most studies support a more significant improvement over 30 days of treatment, so we suggest once every other day with a follow-up of more than 60 days.

Thirdly, with the evaluation methods for neurosensory disorder, many studies considered that there was no statistically significant change in thermal perception test, therefore, we suggest mainly detect outcomes including VAS, TPD, Semmes-weinstein monofilaments, brush stroke and pinprick. The main difficulty focus on the unity of detection methods and the presentation methods and units of the results. Of course, these are all subjective evaluations. It would be more persuasive if the objective indicators was applied, such as trigeminal evoked potential, electrical thermography, electromyography.

Finally, we hope that there will be studies of larger sample size in the future, after all, small sample size studies might exist sampling error and instability. Especially when the random effects model is adopted, the weight of sample size difference is added to increase heterogeneity, which may lead to the deviation of results.

## Conclusion

The results of this META-analysis show that PBM therapy is effective in the treatment of IAN dysfunction caused by orthognathic surgeries, teeth extraction and mandibular fractures no matter it begins early or later. However, due to the limitations number of well-designed RCTs and small number of patients in each study, it would be necessary to conduct randomized controlled trials with large sample size, long follow-up time and more standardized treatment and evaluation methods in the future to provide more accurate and clinically meaningful experimental results.

## Supporting information

S1 Checklist PRISMA 2020 checklist. (DOCX)

S1 Data Minimal data set. (XLSX)
